# Sublethal enteroviral infection exacerbates disease progression in an ALS mouse model

**DOI:** 10.1186/s12974-022-02380-7

**Published:** 2022-01-12

**Authors:** Yuan Chao Xue, Huitao Liu, Yasir Mohamud, Amirhossein Bahreyni, Jingchun Zhang, Neil R. Cashman, Honglin Luo

**Affiliations:** 1 Centre for Heart and Lung Innovation, St. Paul’s Hospital, Vancouver, BC Canada; 2 Department of Pathology and Laboratory Medicine, University of British Columbia, Vancouver, BC Canada; 3 Department of Experimental Medicine, University of British Columbia, Vancouver, BC Canada; 4 Djavad Mowafaghian Centre for Brain Health, University of British Columbia, Vancouver, BC Canada

**Keywords:** Enterovirus, ALS, Neuroinflammation, SOD1 mouse model, TDP-43 pathology

## Abstract

**Background:**

Amyotrophic lateral sclerosis (ALS) is a fatal neurodegenerative disease of the motor neuron system associated with both genetic and environmental risk factors. Infection with enteroviruses, including poliovirus and coxsackievirus, such as coxsackievirus B3 (CVB3), has been proposed as a possible causal/risk factor for ALS due to the evidence that enteroviruses can target motor neurons and establish a persistent infection in the central nervous system (CNS), and recent findings that enteroviral infection-induced molecular and pathological phenotypes closely resemble ALS. However, a causal relationship has not yet been affirmed.

**Methods:**

Wild-type C57BL/6J and G85R mutant superoxide dismutase 1 (SOD1^G85R^) ALS mice were intracerebroventricularly infected with a sublethal dose of CVB3 or sham-infected. For a subset of mice, ribavirin (a broad-spectrum anti-RNA viral drug) was given subcutaneously during the acute or chronic stage of infection. Following viral infection, general activity and survival were monitored daily for up to week 60. Starting at week 20 post-infection (PI), motor functions were measured weekly. Mouse brains and/or spinal cords were harvested at day 10, week 20 and week 60 PI for histopathological evaluation of neurotoxicity, immunohistochemical staining of viral protein, neuroinflammatory/immune and ALS pathology markers, and NanoString and RT-qPCR analysis of inflammatory gene expression.

**Results:**

We found that sublethal infection (mimicking chronic infection) of SOD1^G85R^ ALS mice with CVB3 resulted in early onset and progressive motor dysfunction, and shortened lifespan, while similar viral infection in C57BL/6J, the background strain of SOD1^G85R^ mice, did not significantly affect motor function and mortality as compared to mock infection within the timeframe of the current study (60 weeks PI). Furthermore, we showed that CVB3 infection led to a significant increase in proinflammatory gene expression and immune cell infiltration and induced ALS-related pathologies (i.e.*,* TAR DNA-binding protein 43 (TDP-43) pathology and neuronal damage) in the CNS of both SOD1^G85R^ and C57BL/6J mice. Finally, we discovered that early (day 1) but not late (day 15) administration of ribavirin could rescue ALS-like neuropathology and symptoms induced by CVB3 infection.

**Conclusions:**

Our study identifies a new risk factor that contributes to early onset and accelerated progression of ALS and offers opportunities for the development of novel targeted therapies.

**Supplementary Information:**

The online version contains supplementary material available at 10.1186/s12974-022-02380-7.

## Background

Amyotrophic lateral sclerosis (ALS) is a heterogeneous disease that is now considered to be caused by a combination of genetic and environmental risk factors [1, 2]. To date, more than 40 genes have been reported to be highly associated with ALS, which include *chromosome 9 open-reading frame 72,* (*C9orf72), superoxide dismutase 1* (*SOD1*), and *TAR DNA-binding protein* (*TARDBP*) [3–5]. On the other hand, several environmental factors including viral and bacterial infections, physical activity, smoking, and exposures to heavy metals, pesticides, and chemicals have been suggested as risk factors for ALS, but none have been confirmed [6, 7].

Similar to carcinogenesis, the development of ALS was proposed to be a multistep process involving interactions between genetic mutations and environmental risk factors [1, 2]. This hypothesis places a high interest in exploring the role of environmental factors including viral infection in the development of ALS. Over the past 40 years, efforts have been made to study the association of ALS with different neurotropic viruses, including enteroviruses (EVs) and retroviruses [8, 9]. Among them, human endogenous retroviruses (HERVs) have been widely investigated using clinical diagnostic data, and through in vitro and in vivo experimentations [8, 10–12]. There is evidence that HERV-K gene expression and reverse transcriptase activity can be detected in the blood and brain tissues of some ALS patients. Transgenic mice expressing HERV-K in their neurons display ALS-like motor dysfunction, suggesting a possible role for HERV in ALS pathogenesis [12]. Studies are underway to examine the therapeutic potential of anti-retroviral drugs for ALS (ClinicalTrials.gov Identifier: NCT02437110) [13, 14].

Strong evidence supporting the link between ALS and EV infection is still lacking [9]. EVs are a group of single, positive-stranded RNA viruses of the *Picornaviridae* family that include poliovirus and non-polioviruses, such as coxsackievirus B3 (CVB3), echovirus, EV-A71, and EV-D68 [15, 16]. Although EVs commonly cause asymptomatic infections in adults, infection with EVs can lead to severe neurological complications, such as poliomyelitis, aseptic meningitis, encephalitis, and non-polio flaccid paralysis in children [15, 16]. It has been reported that EVs can persistently exist in various tissues, including the central nervous system (CNS), and reactivate either spontaneously or in response to external stimulations [17–19]. As EVs can target motor neurons, multiple clinical studies have been conducted to explore the possible role of EV infection in ALS [20–27]. However, all these previous studies have been limited to viral genome detection in human ALS blood and tissues, and the available data are controversial and correlative in nature [9]. There is a lack of careful investigation of the potential causal relationship between EV infection and ALS.

Given the possible limitations/challenges, such as differences in methodology for viral detection and the stage of the disease when samples were harvested [9], human viral interrogation study may be unable to provide definitive answers beyond the uncertainty thus far known. Therefore, in the current study, we utilized animal models to determine whether EV infection induces ALS-like phenotype in normal mice and/or promotes early onset and progression of ALS in genetically susceptible mice. We also investigated if administration of anti-EV drugs can alleviate virus-mediated ALS-like symptoms. We demonstrated that sublethal CVB3 infection in an ALS mouse model expressing a human mutant SOD1, SOD1^G85R^, accelerates disease progression and decreases mouse survival. This observation was accompanied by significantly increased immune cell infiltration and proinflammatory cytokine/chemokine gene expression and detection of tissue damage and transactive response DNA binding protein-43 (TDP-43) pathology in the CNS. Moreover, we discovered that application of anti-viral drug ribavirin during the acute phase of viral infection rescues virus-mediated ALS-like pathology and phenotype in mice.

## Methods

### Mice, virus and ribavirin inoculations

SOD1^G85R^ (B6.Cg-Tg (SOD1^G85R^) 148Dwc/J) and its background strain C57BL/6J mice were obtained from Jackson Laboratory (Maine, United States). The neonates (2–3 days) of SOD1^G85R^ or C57BL/6J mice were intracerebroventricularly injected at the intersection between the superior sagittal sinus and the transverse sinus with either a non-lethal dose (5 × 10^2^ pfu (plaque-forming units) in 2 μl volume) of CVB3 (Kandolf strain) or an equal volume of Dulbecco's modified Eagle’s medium (DMEM, mock infection). The viral dosage was selected based on our pilot study that tested multiple viral dosages. This amount of virus did not cause virus-related mortality in C57BL/6J mice despite the virus persisting in the CNS for months.

Both female and male mice were used for the current study. For survival and motor function analysis, the total animal numbers for each group including numbers of male vs female mice are specified in figure legends (unequal number of males vs females was due to a higher ratio of female to male offspring observed during breeding). Mice that developed hydrocephalus after CVB3/DMEM injection or non-ALS/CVB3-related symptoms including developmental abnormality were excluded from this study. For gene expression and immunohistochemistry studies, different cohort of mice (3–5 mice for each timepoint and each group) was used. All animal procedures were approved by the University of British Columbia Research Ethics Board (Animal Care #A20-0156 and A17-0227).

Ribavirin (Sigma-Aldrich, St. Louis, MO; R9644) treatment in CVB3-infected SOD1^G85R^ mice was performed by subcutaneously injecting either ribavirin (100 mg/kg mouse body weight) diluted in 100 μl of DMEM or an equal volume of DMEM in the neck of neonatal mice. The injections were done every 3 days starting at either day 1 or day 15 post-infection (PI) for a total of 5 injections.

### Mouse motor function tests

After 20 weeks of mock or CVB3 inoculation, SOD1^G85R^ (B6.Cg-Tg (SOD1^G85R^) 1248Dwc/J) and C57BL/6J mice underwent weekly motor function tests (hindlimb reflex, inverted grid test, and gait analysis) until either reaching humane endpoint or 60 week post-infection [28, 29]. The motor function measurement was conducted together by two well-trained raters who were blind to the experimental conductions.

Hindlimb reflex was performed by lifting the mice from the tip of the tail to assess and score their hind leg reflex on a scale of 4–0 (4 = healthy animal with full extension of both hind legs, and 0 = both limbs are fully clasped). Reduction in leg extension is an early deficit observed in mutant SOD1 transgenic mice.

An inverted grid test was performed by placing the mice on an inverted metal grid (10 cm above the surface) and measuring the time (in seconds, with the maximum monitoring time as 120 s) for the mice to fall. The test is used to assess the arm/leg strength of the mice [30].

Gait analysis is a test that quantifies the mouse movement based on its gait distance. In the assessment, the mouse feet were first marked by non-toxic paints and then placed on a long paper (60 cm long, 20 cm wide) that was covered by cardboard, allowing the mice to walk forward in one direction. After the paints were dried, the stride distance (the distance of forward movement between each stride) was measured and recorded. In addition to the gait measurements, the time in seconds (footprint time) needed for each mouse to walk over the track was recorded as an additional measurement of the animal’s motor function [31].

### Tissue preparation and immunohistochemistry (IHC) staining

Mouse brains harvested were fixed with 4% formaldehyde and paraffinized. Paraffin-embedded Sections (4 µm thickness) were deparaffinized through xylene and a series of isopropanol solutions (100%, 90%, and 70%). Hematoxylin and eosin (H&E) staining was conducted to evaluate tissue damages, while IHC staining was used for detecting the presence and localization of proteins of interest. For IHC, antigen retrieval was done by heating tissue sections in pH 6.0 citrate buffer (Life Technologies Carlsbad, CA) for 25 min at 121 °C, and then peroxidase blocked using 30 mg/ml hydrogen peroxide solution. Multiple washes with 1 × TBS pH 7.6 (Tris-buffered Saline, 0.05 M Tris, 0.155 M NaCl) were performed afterward. The MACH4 Universal HRP-Polymer Detection System (Biocare Medical, Pacheco CA) was used according to the manufacture’s procedure for IHC staining experimentations. After the TBS washes, tissue sections were incubated with primary antibodies (dilution and targets are shown below) overnight at 4 °C, and counterstained at the end with hematoxylin solution Gill II (Sigma-Aldrich, St. Louis, MO).

Mouse brain tissues were immunostained using the follow primary antibodies diluted in Tris-buffered saline (pH 7.6) containing 1% bovine serum albumen: VP1 (1:2000 dilution, IgG2a monoclonal antibody, Mediagnost, Reutlingen, Germany), GFAP (1:200 dilution, mouse monoclonal antibody, StressMarq, Victoria, BC, Canada), Iba1 (1:200 dilution, mouse monoclonal antibody, Santa Cruz Biotechnology, Dallas, TX, USA), TDP-43 (1:1000 dilution, rabbit polyclonal antibody, Proteintech, Rosemont, IL, USA), SQSTM1/p62 (1:200 dilution, mouse monoclonal antibody, Santa Cruz Biotechnology, Dallas, TX, USA), ubiquitin (1:2000 dilution, mouse monoclonal antibody, Santa Cruz Biotechnology, Dallas, TX, USA), CD68 (1:200 dilution, mouse monoclonal antibody, Santa Cruz Biotechnology, Dallas, TX, USA), CD19 (1:500 dilution, mouse monoclonal antibody, Santa Cruz Biotechnology, Dallas, TX, USA), CD79A (1:300 dilution, mouse monoclonal antibody, Santa Cruz Biotechnology, Dallas, TX, USA), CD4 (1:500 dilution, mouse monoclonal antibody, Santa Cruz Biotechnology, Dallas, TX, USA), CD8 (1:500 dilution, mouse monoclonal antibody, Santa Cruz Biotechnology, Dallas, TX, USA), and NK1.1 (1:1000 dilution, mouse monoclonal antibody, eBioscience, San Diego, CA, USA). Images were taken using the Aperio ScanScope AT (Digital slide scanner, Leica Biosystems Inc., Buffalo Grove, IL, USA).

### RNA extraction and gene expression assays

Mouse brain and spinal cord tissues were homogenized using Qiagen Tissuelyzer LT, and RNAs were extracted using the Monarch total RNA miniprep kit (New England Biolabs) following the manufacturer’s instructions. Gene expression was measured using NanoString Mouse Immunology Panel (561 targets with 15 internal reference targets) that was ran on a NanoString nCounter® Profiler (NanoString Technologies Inc., Seattle, WA, USA) according to the manufacturer’s instructions. NanoString data were analyzed using Gene Ontology and statistical difference in gene expression between mock- and CVB3-infected SOD1^G85R^ was determined by unpaired student’s *t* test (*p* < 0.05 is considered statistically significant). The mRNA levels of different proinflammatory genes and viral RNA were measured via RT-qPCR using the Luna universal one-step RT-qPCR kit (New England Biolabs) on a QuantStudio 6 Pro real-time PCR system (Applied Biosystems, Foster City, CA, USA). The primer pairs used for mRNA measurement in mouse tissues are as follows: *TNFA* (forward, 5*’-* GTC CCC AAA GGG ATG AGA AGT T -3’; reverse, 5’- GTT TGC TAC GAC GTG GGC TAC A -3’), *NFKB2* (forward, 5*’-* TGC TGA TGG CAC AGG ACG AGA A -3’; reverse, 5’- GTT GAT GAC GCC GAG GTA CTG A -3’), *CXCL10* (forward, 5*’-* GCT GGG ATT CAC CTC AAG AA -3’; reverse, 5’- CTT GGG GAC ACC TTT TAG CA -3’), *CCL2* (forward, 5*’-* GCT ACA AGA GGA TCA CCA GCA G -3’; reverse, 5’- GTC TGG ACC CAT TCC TTC TTG G -3’), *CCL5* (forward, 5*’-* GCT TTG CCT ACC TCT CC -3’; reverse, 5’- TCG AGT GAC AAA CAC GAC TGC -3’), *IL6* (forward, 5*’-* ACA ACC ACG GCC TTC CCT AC -3’; reverse, 5’- TCT CAT TTC CAC GAT TTC CCA G -3’), *IL10* (forward, 5*’-* CCC ATT CCT CGT CAC GAT CTC -3’; reverse, 5’- TCA GAC TGG TTT GGG ATA GGT TT -3’), *CVB3 2A* (forward, 5′-GCT TTG CAG ACA TCC GTG ATC-3′; reverse, 5′-CAA GCT GTG TTC CAC ATA GTC CTT CA-3′), and *β-actin* (forward, 5*’-* CAT TGC TGA CAG GAT GCA GAA GG -3’; reverse, 5’- TGC TGG AAG GTG GAC AGT GAG G -3’).

### Quantification and statistical analysis

Quantification of IHC images was performed using ImageJ (version 1.52p) with the combination of Colour Deconvolution Plugin as described [32]. The optical density (OD) value was calculated based on the intensity of the IHC staining using the formula: OD = log (max intensity/mean intensity), where the max intensity = 255. Data generated were from at least three separate images from the hippocampus and cerebral cortex regions for each mouse. Statistical analysis and the corresponding graphs were performed using GraphPad Prism V8.4. Further details in statistical analysis are described in figure legends.

## Results

### Sublethal CVB3 infection exacerbates ALS-like phenotypes and decreases survival of ALS SOD1^G85R^ mice

**Fig. 1:**
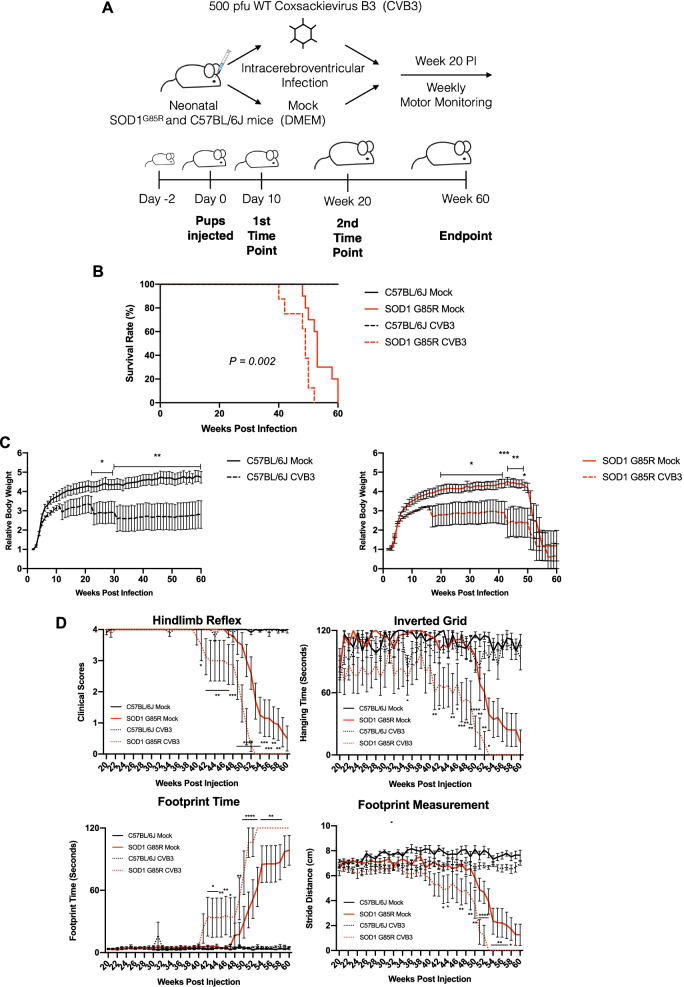
Sublethal CVB3 infection exacerbates ALS-related phenotypes and decreases the lifespan of SOD1^G85R^ mice. **A** Schematic diagram illustrating intracerebroventricular injections of neonatal SOD1^G85R^ and C57BL/6J mice with either 500 pfu of CVB3 or an equal volume of DMEM (mock infection). Mouse motor functions were monitored weekly starting at week 20 post-infection (PI) until the mice were approaching the humane endpoint or at the experimental endpoint of week 60 PI. Mouse tissues were collected at day 10, week 20 and week 60 PI or at the humane endpoint. **B** Kaplan–Meier plots comparing survival rates among C57BL/6J Mock (*n* = 12; male = 4 and female = 8), SOD1^G85R^ Mock (*n* = 10; male = 3 and female = 7); C57BL/6J CVB3 (*n* = 9; male = 4 and female = 5), and SOD1^G85R^ CVB3 (*n* = 8; male = 2 and female = 6) mice. *P* = 0.002 between SOD1^G85R^ mice inoculated with CVB3 and mock-infected. **C** Relative body weight measured weekly after infection. Body weight at day 0 was arbitrarily set to 1. **D** Mouse motor function measurement, including hindlimb reflex score, inverted grid time, footprint time, and stride distance, assessed weekly after infection. Quantifications are presented as mean ± standard error of the mean (SEM). Statistical analysis was carried out by two-way ANOVA, followed by Bonferroni’s multiple comparison test. *, *p* < 0.05; **, *p* < 0.01; ***, *p* < 0.001; ****, *p* < 0.0001

To determine the impact of EV infection on the development of ALS, the neonates (2–3 days) of transgenic mice carrying human mutant SOD1 (SOD1^G85R^) and non-transgenic C57BL/6J (background strain used as experimental control) mice were intracerebroventricularly inoculated with a sublethal dose of CVB3 (500 pfu) or an equal volume of DMEM (mock infection) for 10 days, 20 weeks or 60 weeks (Fig. 1A). The viral dosage was chosen based on our previous experience in non-transgenic mice, which does not cause virus-related mortality despite the virus persisting in the CNS for months. Weekly motor function measurements were performed by hindlimb reflex score, inverted grid test, and gait analysis starting at week 20 post-infection (PI). We selected the SOD1^G85R^ model, because these mice develop a slowly progressive and late adult-onset paralysis [33], unlike other models of ALS, such as SOD1^G93A^ mice, which rapidly develop paralysis [34], obscuring the role of EV infection in ALS. In addition, the SOD1^G85R^ mice express levels of *SOD1*^*G85R*^ equivalent to those of endogenous *SOD1*, thus closely mimicking human cases of ALS.

We showed that sublethal CVB3 infection led to a significantly shortened lifespan of SOD1^G85R^ mice as compared to mock-infected SOD1^G85R^ mice (average 47.5 weeks vs 53.6 weeks, *p* = 0.002) (Fig. 1B). Decreased body weight was also observed in CVB3-infected SOD1^G85R^ mice starting at around week 19 PI compared with mock-infected SOD1^G85R^ mice (Fig. 1C, right). While CVB3 did not cause mortality in C57BL/6J mice (Fig. 1B), the body weight in CVB3-infected C57BL/6J mice was lower than that in mock-infected counterparts (Fig. 1C, left). Motor function measurements revealed a significant decrease in hindlimb reflex score, hanging time, and stride distance, as well as a drastic increase in footprint time in SOD1^G85R^ mice after CVB3 infection starting around week 40 PI compared with mock infection (Fig. 1D). It was observed that CVB3 infection of C57BL/6J mice did not trigger motor dysfunction (Fig. 1D). Altogether, these results suggest that sublethal CVB3 infection accelerates disease onset, exacerbates motor dysfunction, and decreases survival in the SOD1^G85R^ mice but does not seem to cause similar phenotypes in C57BL/6J mice.

It should be noted that both male and female mice were included in this study. However, statistical analysis revealed no significant difference in the changes of motor function, body weight, and survival between males and females in our experimental model (data not shown). Therefore, all results presented here were data from both sexes combined.

### Sublethal CVB3 infection leads to ALS-related protein pathologies in vivo

**Fig. 2:**
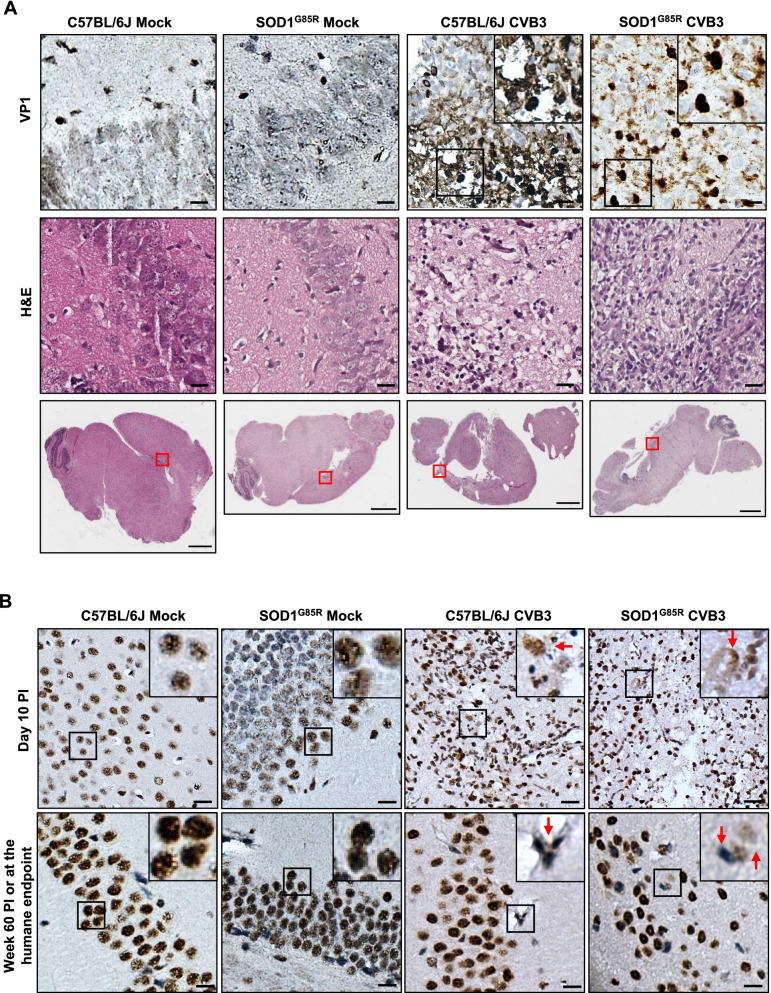
Sublethal CVB3 infection leads to ALS-related pathologies in vivo. **A** Representative images of viral protein VP1 immunohistochemical and H&E histological staining in the hippocampus regions of the brain from mock- and CVB3-infected C57BL/6J or SOD1^G85R^ mice at day 10 PI. The red boxes indicate the region of the magnification. Black boxes on the top right illustrate the enlarged images. **B** Representative images of TDP-43 immunohistochemical staining in the hippocampus regions of the brain from mock- and CVB3-injected C57BL/6J or SOD1^G85R^ mice at day 10 PI or week 60 PI (or humane endpoint) as indicated. The red arrows indicate TDP-43 mislocalization. Black boxes on the top right illustrate the enlarged images. Scale bar of magnified images = 100 μm. Scale bar of whole brain images = 1000 μm

Having shown that CVB3 infection leads to exacerbated disease phenotypes and shortened survival in SOD1^G85R^ mice, we next examined the neuropathological changes in CVB3-infected mice. Similar to our previous observations in Balb/c mice [35], we were able to detect CVB3 capsid protein VP1 in mouse brain at day 10 PI in multiple regions of the brain, including the hippocampus (Fig. 2A, top panel), cerebral cortex, olfactory bulb, striatum, and putamen regions (data not shown). VP1 signals were not detectable in the brain at week 60 PI as expected due to immune-mediated viral clearance (data not shown). H&E staining showed significant tissue damages in the same regions of the mouse brain as VP1 was detected (Fig. 2A, middle panel).

We also examined ALS-related pathologies through IHC staining of TDP-43 (an RNA-binding protein), SQSTM1/p62 (an ubiquitin-binding autophagy adaptor protein), and ubiquitin. Similar to our previous findings [35], we observed that CVB3 infection led to cytoplasmic mislocalization of TDP-43 in both SOD1^G85R^ and C57BL/6J mice at day 10 and week 60 PI (Fig. 2B). In mock-infected mice, TDP-43 remained in the nucleus. Furthermore, SQSTM1/p62- and ubiquitin-positive inclusions were detected in the brain of CVB3-infected but not mock-infected mice at both day 10 and week 60 PI (Additional file 1: Fig. S1). Collectively, these results indicate that CVB3 infection induces ALS-related pathologies (e.g., TDP-43 mislocalization, the pathological hallmark of ALS), which may contribute to the accelerated ALS phenotypes observed in CVB3-infected SOD1^G85R^ mice.

### Sublethal CVB3 infection promotes immune cell infiltration and proinflammatory gene expression

The role of neuroinflammation in the development of neurodegenerative diseases, especially in ALS, has been widely studied and emphasized [36–38]. Therefore, we next sought to investigate the inflammatory response in both SOD1^G85R^ and C57BL/6J mice after CVB3 infection. IHC staining was conducted to assess the astrocyte reactivity with anti-GFAP (glial fibrillary acidic protein) antibody, microglial activation using anti-Iba1 (ionized calcium-binding adaptor molecule 1) and anti-CD68 (cluster of differentiation 68) antibodies, the presence of B cells with anti-CD19 and anti-CD79A antibodies, the presence of T cells using anti-CD4 and anti-CD8 antibodies, and the presence of natural killer (NK) cells with anti-NK1.1 (an NK cell-specific antigen) antibody.

**Fig. 3:**
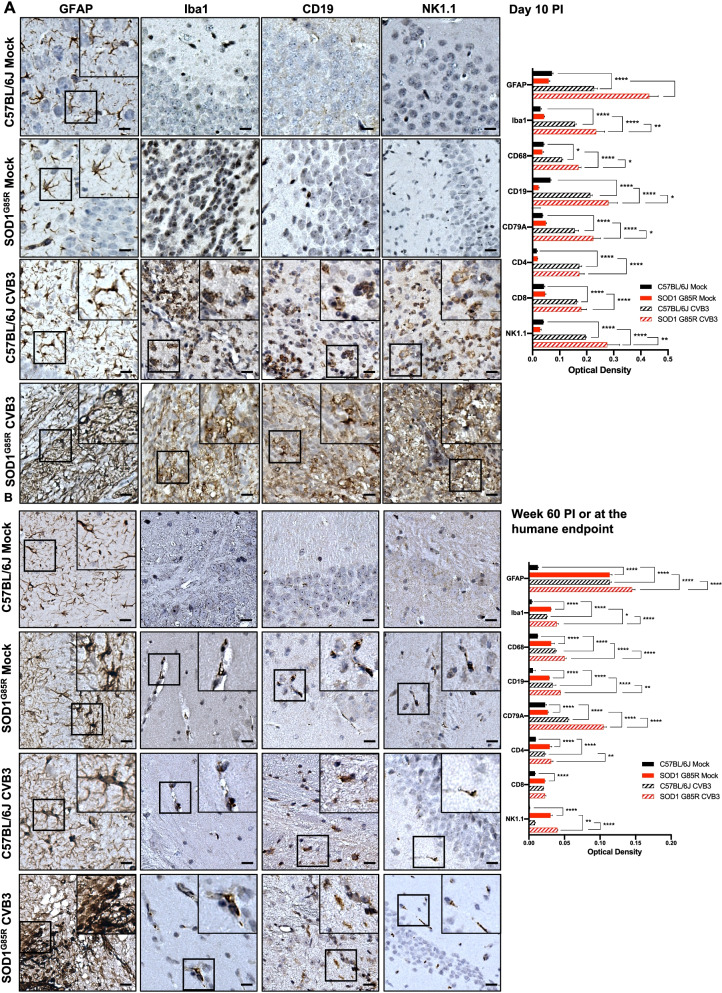
Sublethal CVB3 infection triggers microglia/astrocyte activation and immune infiltration in mice. Representative images of GFAP, Iba1, CD19 and NK1.1 immunohistochemical staining in the hippocampus regions of the brain from mock- or CVB3-infected C57BL/6J or SOD1^G85R^ mice at day 10 PI (**A**) or week 60 PI (or humane endpoint) (**B**). Similar observation was made in the regions of infected cerebral cortex (data not shown). Images for other targets (CD68, CD79A, CD4, can CD8) can be found in the Additional file 2: Fig S2. Immune infiltrations were quantified by optical density based on the IHC staining in the hippocampus and cerebral cortex regions. Black boxes on the top right illustrate the enlarged images. Quantifications are presented as mean ± SEM (*n* = 3–4 individual mouse brain samples for each target, timepoint and experimental condition). Scale bar = 100 μm. Statistical analysis was carried out using two-way ANOVA, followed by Bonferroni’s multiple comparison test. *, *p* < 0.05; **, *p* < 0.01; ***, *p* < 0.001; ****, *p* < 0.0001

As shown in Fig. 3A and Additional file 2: Fig. S2A, low levels of different immune cell markers were observed in mock-infected C57BL/6J and SOD1^G85R^ mice at day 10 PI. However, upon CVB3 infection, both C57BL/6J and SOD1^G85R^ mice demonstrated significant increases in infiltration/activation of astrocytes (GFAP), microglia/macrophages (Iba1 and CD68), B cells (CD19 and CD79A), T cells (CD4 and CD8) and NK cells (NK1.1) in the brain at day 10 PI. At week 60 PI, mock-infected SOD1^G85R^ mice showed significantly higher levels of different immunological markers as compared to mock-infected C57BL/6J mice (Fig. 3B and Additional file 2: Fig. S2B), consistent with previous reports that mutant SOD1 can elicit immune responses [39–41]. It was also found that immunological markers of GFAP, Iba1, CD68, CD19, CD79A, CD4, and NK1.1 were increasingly expressed in the brain of CVB3-infected SOD1^G85R^ mice than virus-infected C57BL/6J mice at week 60 PI. Notably, CVB3 infection of SOD1^G85R^ mice induced an additive expression of immunological markers than either CVB3 infection or SOD1^G85R^ alone at both day 10 and week 60 PI (Fig. 3A, B).

**Fig. 4:**
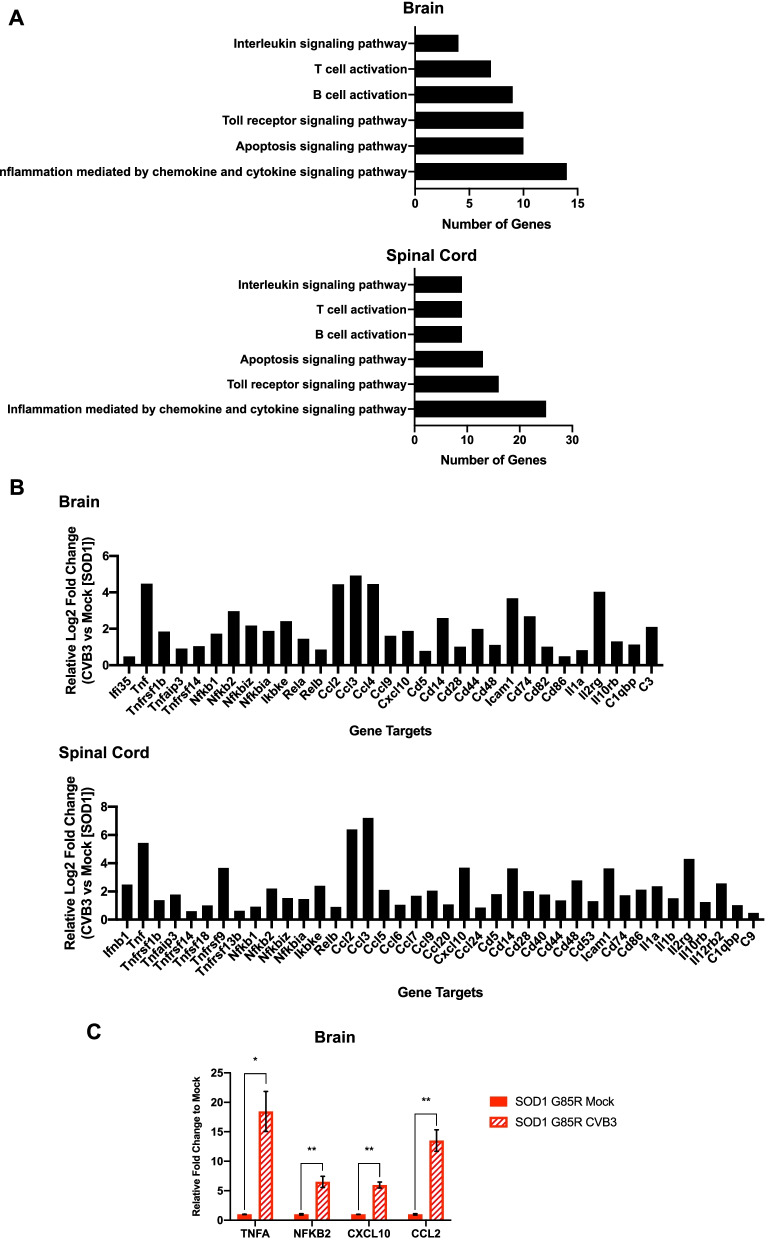
CVB3 infection promotes proinflammatory responses in the CNS of SOD1^G85R^ mice. **A**–**B** Gene expression comparison of different proinflammatory cytokines/chemokines conducted using NanoString mouse immunology panel on the brain and spinal cord samples harvested from mock- (*n* = 3) and CVB3-infected SOD1^G85R^ mice (*n* = 3) at week 20 PI. Panel (**A**) shows the Gene Ontology analysis of significantly expressed genes (*p* < 0.05, via student’s t test) in the brain (top) and spinal cord (bottom) of CVB3-infected SOD1^G85R^ mice normalized to mock-infected counterparts. Panel (**B**) shows differentially expressed cytokine/chemokine genes (in relative Log2 fold changes) in the brain (top) and spinal cord (bottom) of CVB3-infected SOD1^G85R^ mice normalized to mock-infected counterparts. **C** RT-qPCR validation of four significantly increased gene expression of *TNFA, NFKB2, CXCL10* and *CCL2* in the same brain samples (week 20 PI) as above. Quantifications are presented as mean ± SEM (*n* = 3–4 for each group). Statistical analysis was carried out using student’s t test. *, *p* < 0.05; **, *p* < 0.01

We further investigated the alteration of proinflammatory gene expression in both the brain and spinal cord from mock- and CVB3-infected SOD1^G85R^ mice at week 20 PI by NanoString using nCounter mouse immunology panel. Gene Ontology (GO) analysis showed upregulated genes in the brain (105 genes) and spinal cord (141 genes) of CVB3-infected relative to mock-infected SOD1^G85R^ mice, which can be categorized into six different functional groups related to immune responses as indicated (Fig. 4A). A closer examination of these significantly upregulated genes revealed that many critical proinflammatory cytokine/chemokine genes were present in both the brain and spinal cord samples (Fig. 4B). RT-qPCR analysis confirmed the upregulation of four genes (*TNFA*, *NFKB2*, *CXCL10,* and *CCL2*), which are shared between the brain and spinal cord samples, in the brain of week 20 CVB3-infected compared to mock-infected SOD1^G85R^ mice (Fig. 4C). Taken together, our results indicate that CVB3 infection further enhances immune cell infiltration and proinflammatory gene expression in SOD1^G85R^ mice.

### CVB3-accelerated disease progression is mitigated by early viral intervention

**Fig. 5:**
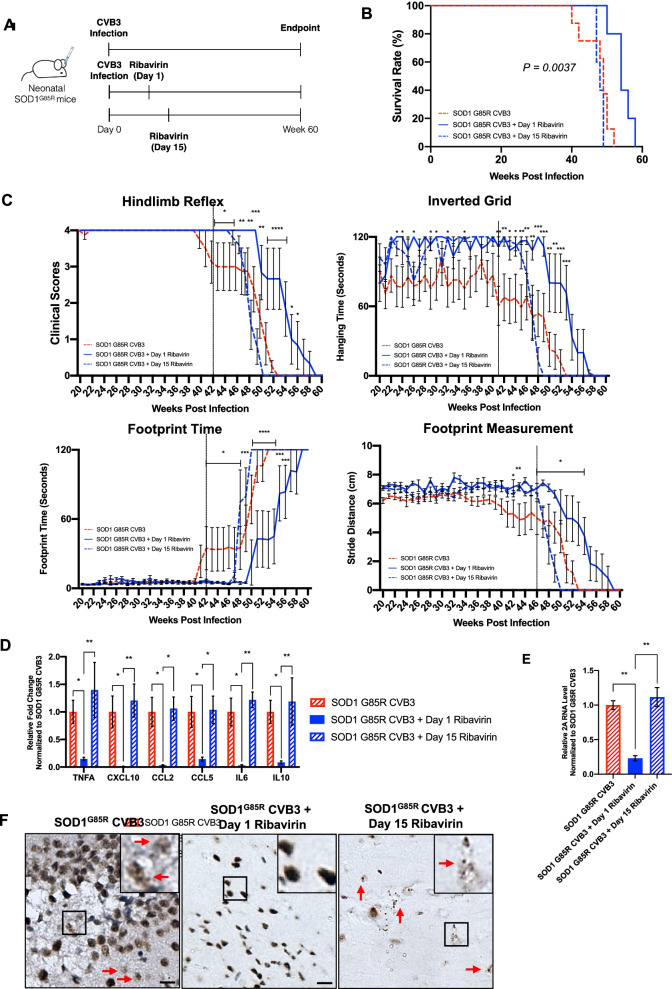
CVB3-accelerated ALS progression is mitigated by early antiviral treatment. **A** Schematic diagram illustrating the experimental plan for CVB3 injection, ribavirin administration, and the endpoint. **B** Kaplan–Meier plots comparing mouse survivals among SOD1^G85R^ CVB3 (*n* = 8; male = 2 and female = 6), SOD1^G85R^ CVB3 day 1 ribavirin (*n* = 5; male = 2 and female = 3), and SOD1^G85R^ CVB3 day 15 ribavirin (*n* = 5; male = 2 and female = 3) mice. *P* = 0.0037 between SOD1^G85R^ CVB3 and SOD1^G85R^ CVB3 day 1 ribavirin groups. **C** Mouse motor function measured weekly (i.e., hindlimb reflex score, inverted grid time, footprint time, and stride distance) starting at week 20 PI until humane or experimental endpoint. The vertical black line indicates the point of deviation between the two groups. **D**–**E** RT-qPCR evaluation of proinflammatory genes (**D**) and viral RNA (**E**) in the brain tissues collected at week 60 PI or the humane endpoints. **F** Representative TDP-43 IHC staining images in the hippocampus regions of the brain collected at week 60 PI or the humane endpoint. The red arrows indicate TDP-43 mislocalization. Scale bar = 100 μm. Quantifications are presented as mean ± SEM (*n* = 3–4). Statistical analysis was carried out by two-way ANOVA, followed by Bonferroni’s multiple comparison test. *, *p* < 0.05; **, *p* < 0.01

Finally, we examined whether administration of ribavirin, a broad-spectrum anti-RNA viral drug, during the acute or chronic stage of infection can rescue virus-mediated ALS-like neuropathology and symptoms. Ribavirin is a nucleoside analog and acts as a mutagen via incorporation into the viral RNA genome. It can cross the blood–brain barrier and inhibit neurotropic replication of a variety of EVs [16, 42]. Ribavirin (100 mg/kg body weight), diluted in DMEM, was subcutaneously injected to CVB3-infected SOD1^G85R^ mice every 3 days starting at either day 1 (early intervention) or day 15 (late intervention) PI for a total of 5 injections (Fig. 5A). This amount of ribavirin has been previously shown to inhibit CVB3 infection in the CNS [43]. After the last injection, all mice underwent similar experimental procedures as illustrated in Fig. 1A. Kaplan–Meier plots of mouse survival showed that early intervention with ribavirin significantly extended mouse survival compared with CVB3-infected SOD1^G85R^ mice receiving no treatment (average lifespan of 54.4 weeks vs 47.5 weeks, *p* = 0.0037) (Fig. 5B). However, late intervention with ribavirin did not cause improvement of mouse survival (average lifespan of 48.0 weeks vs 47.5 weeks).

A significant improvement in the motor function was observed in CVB3-infected SOD1^G85R^ mice after early rather than late intervention with ribavirin (Fig. 5C). Immunologically, there was a significantly lower expression of proinflammatory genes, including *TNFA, CXCL10, CCL2, CCL5, IL6,* and *IL10*, in the early but not late treatment group as compared to the non-treated group (Fig. 5D). We also measured viral RNA levels in the brain and showed that early but not late ribavirin treatment significantly decreased the level of viral RNA (Fig. 5E). Finally, we were able to show TDP-43 mislocalization in the late, but not in the early intervention group (Fig. 5F). Jointly, the results suggest that application of anti-EV drugs at the early stage of infection attenuates ALS-like phenotype and improves animal survival.

## Discussion

The rationale for the current study stems from early evidence that EVs can target motor neurons and from the recent exciting discovery that EV infection produces the hallmark molecular phenotypes of ALS [9], including neuroinflammation, RNA-processing defects, compromised protein quality control and protein aggregation, impaired nucleocytoplasmic transport, and most intriguingly, cytoplasmic mislocalization, aggregation, and cleavage of TDP-43, termed TDP-43 pathology [35, 44]. TDP-43 pathology, found in more than 95% of all deceased ALS patients, is not only a pathological hallmark of ALS but also a key disease mechanism for ALS [45]. Despite these exciting observations, a compelling causal relationship between EV infection and ALS development has not yet been established.

**Fig. 6:**
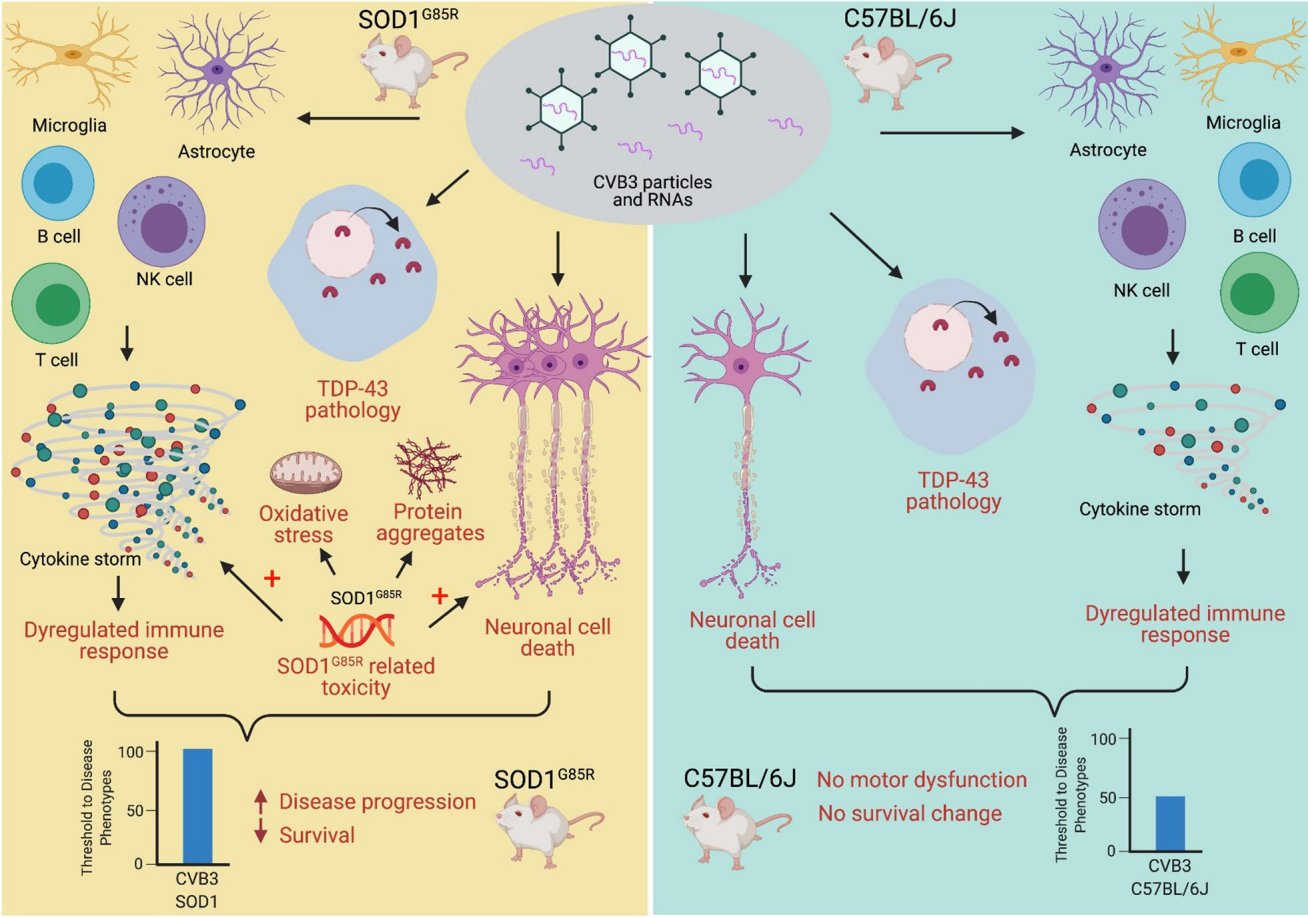
Schematic summary of mechanisms that enteroviral infection exacerbates disease phenotype in an ALS mouse model. Sublethal infection of enteroviruses, such as CVB3 modelled in this study, promotes early onset and progression of ALS-like phenotype, and decreases the lifespan of mice in SOD1^G85R^ ALS mouse model. This exacerbation is associated with at least three molecular and pathological phenotypes induced by CVB3 infection: (1) direct tissue damages or neuronal cell death, (2) dysregulated immune responses, and (3) TDP-43 pathologies. It appears that sublethal viral infection alone is not sufficient to provoke ALS-like phenotypes in C57BL/6J mice. However, together with SOD1^G85R^-mediated toxicities (e.g., increase proinflammatory response, oxidative stress, protein aggregates and neuronal cell death), viral infection accelerates disease progression and reduces mouse lifespan in SOD1^G85R^ ALS mice

Utilizing mouse models, in the present study we demonstrated that chronic, postnatal CVB3 infection hastens disease onset, accelerates motor dysfunction, and shortens the lifespan of SOD1^G85R^ mice, while similar sublethal viral infection is unable to elicit an ALS-like phenotype in normal non-transgenic mice at least within the timeframe of this study (60 weeks PI). Overall, our results suggest that EV infection serves as a risk/susceptibility factor, rather than a cause for ALS. We propose that at least three mechanisms contribute to the exacerbating effects of EV infection on ALS onset and progression (Fig. 6).

First, in line with previous reports [17, 35, 43], we showed here that CVB3 infection of the CNS causes focal damages and viral protein expression in multiple regions of the brain, including the hippocampus, cerebral cortex, olfactory bulb, striatum, and putamen, which can lead to potential behavior and motor function changes.

Second, we observed that immunological markers and proinflammatory gene expression are upregulated in the CNS of mice expressing mutant SOD1 and CVB3 infection further enhances immune cell infiltration/activation and cytokine/chemokine gene expression. Neuroinflammation has been identified as a key player in the pathogenesis of ALS [36–38]. The evidence presented here suggested an important mechanism by which EV infection worsens ALS-linked phenotypes through promoting aberrant immune responses. We demonstrated the presence of several immune cell types, such as macrophages, T cells, B cells, and NK cells within the brain of CVB3-infected C57BL/6J and SOD1^G85R^ mice or mock-infected SOD1^G85R^ mice at week 60 PI. Notably, the infiltration of these immune cells is more evident at day 10 PI compared with week 60 PI in both CVB3-infected C57BL/6J and SOD1^G85R^ mice, indicating a key role of the early phase immune cell infiltration in CVB3-induced disease progression. Likewise, the expression level of different immune mediators in the brain was higher in the CVB3-infected SOD1^G85R^ mice as compared to mock-infected SOD1^G85R^ mice at week 20 PI, supporting the notion that early CVB3 infection kickstarts immune dysregulation. While there is evidence supporting the involvement of macrophage, T and NK cells in the development of ALS [46–49], the role of B cells appears to be insignificant when comparing the disease progression of SOD1^G93A^ mice with that of SOD1^G93A^ mice deficient of B cells [50]. However, in our experimental model, we found increased B cell infiltration in CVB3-infected SOD1^G85R^ mice as compared to other conditions, suggesting a role for B cells in disease development during virus infection possibly via antibody-dependent and/or -independent mechanisms [51].

Third, similar to our previous report [35], we observed ALS-related pathologies (TDP-43 mislocalization, SQSTM1/p62- and ubiquitin-positive inclusions) within CVB3-infected mouse brains. TDP-43 pathology is not generally detectable in SOD1-related ALS animal models and human tissues with SOD1 mutations [52, 53]. Therefore, CVB3-induced TDP-43 mislocalization likely serves as another mechanism contributing to accelerated disease progression,

It is noted that, despite shared pathological and molecular characteristics as that in CVB3-infected SOD1^G85R^ mice, such as virus-induced tissue damages, increase immune cell infiltration, and positive ALS-related pathologies, CVB3 infection of the C57BL/6J background strain mice is not sufficient in inducing motor dysfunction and decreasing survival. We speculate that virus-induced tissue damages, molecular pathologies, and immune responses, under a healthy genetic background, could be tolerated by the host and eventually surmounted (i.e., not reaching the threshold for disease phenotype). However, similar damages/immune responses in combination under a detrimental genetic mutation background, such as SOD1^G85R^, could contribute to the acceleration of disease progression (Fig. 6).

Finally, as a proof-of-concept, we tested whether the application of an anti-EV drug can attenuate ALS phenotype and improve animal survival. To further understand how CVB3 infection affects the disease progression in SOD1^G85R^ mice, we treated CVB3-infected mice with ribavirin at either day 1 or day 15 PI. By comparing the results of early vs late treatment, we gain a better understanding of the question whether neurodegeneration will continue if viral infection is stopped and the possible role of “prion-like mechanism” in ALS [54, 55]. For example, less effectiveness of late treatment compared with early treatment may suggest a “prion-like mechanism” independent of persistent viral infection for disease progression. In other words, persistent/active infection would not necessarily be required for disease progression. In this study, we demonstrated that early intervention with ribavirin mitigates disease progression and improves mouse survival, whereas late intervention fails to provide a protective effect, suggesting a potential involvement of a “prion-like mechanism” in EV-related ALS. Another contributing factor to this blockage of TDP-43 pathology and mitigation of worsened disease progression in the early vs late treatments is the significant decrease in immune responses as evidenced by the decrease of gene expression in several proinflammatory cytokines after day 1 but not day 15 ribavirin treatment. The evidence suggests that the timing of intervention is critical in the disease development and that virus reduction in the early phase of infection could prevent aberrant immune activation. Future studies are warranted to examine the role of a combination of both anti-viral and anti-propagation drugs in this model.

## Conclusions

In this study, we identified EV as a novel risk factor for the initiation and progression of ALS, which offers potential for future therapeutic interventions. Knowledge gathered from this project will also have broad implications for the study of host–pathogen interactions in other neurodegenerative diseases, in particular frontotemporal dementia, which share many common neuropathological hallmarks and disease mechanisms with ALS [56, 57].

## Supplementary Information

**Additional file 1: Fig. S1.** Sublethal CVB3 infection leads to ALS-related pathologies in vivo*.* Representative images of SQSTM1/p62 and ubiquitin immunohistochemical staining in the hippocampus regions of the brain from mock- and CVB3-injected C57BL/6J or SOD1^G85R^ mice at day 10 PI or week 60 PI (or humane endpoint) as indicated. The red arrows indicate SQSTM1/p62 or ubiquitin positive inclusions. Black boxes on the top right illustrate the enlarged images. Scale bar = 100 μm.

**Additional file 2: Fig. S2.** Sublethal CVB3 infection triggers microglia/astrocyte activation and immune infiltration in mice. Representative images of CD68, CD79A, CD4 and CD8 immunohistochemical staining in the hippocampus regions of the brain from mock- and CVB3-infected C57BL/6J or SOD1^G85R^ mice at day 10 PI **(A)** or week 60 PI (or humane endpoint) **(B)**. Similar observation was made in the regions of infected cerebral cortex (data not shown). Immune infiltrations were quantified by optical density based on the IHC staining in the hippocampus and cerebral cortex regions. Black boxes on the top right illustrate the enlarged images. Scale bar = 100 μm.

## Data Availability

The data supporting the findings of this study are available from the corresponding author upon request.

## Abbreviations

ALS: Amyotrophic lateral sclerosis
C9orf72: Chromosome 9 open-reading frame 72
CCL2/5: C–C motif chemokine ligand 2/5
CD4/8/19/68/79A: Cluster of differentiation 4/8/19/68/79A
CNS: Central nervous system
CVB3: Coxsackievirus type B3
CXCL10: C–X–C motif chemokine ligand 10
DMEM: Dulbeccco’s modified Eagle’s medium
EV: Enterovirus
GFAP: Glial fibrillary acidic protein
GO: Gene ontology
H&E: Hematoxylin and eosin
HERV: Human endogenous retrovirus
Iba1: Ionized calcium binding adaptor molecule 1
IHC: Immunohistochemistry
IL6/10: Interleukin 6/10
NFκB: Nuclear factor kappa B
NK: Natural killer
OD: Optical density
pfu: Plaque-forming units
PI: Post-infection
SOD1: Superoxide dismutase 1
SQSTM1: Sequestosome 1
TARDBP/TDP-43: TAR DNA-binding protein/ TAR DNA-binding protein 43
TNFA: Tumor necrosis factor alpha

## References

[1] Al-Chalabi A, Calvo A, Chio A, Colville S, Ellis CM, Hardiman O, Heverin M, Howard RS, Huisman MHB, Keren N (2014). Analysis of amyotrophic lateral sclerosis as a multistep process: a population-based modelling study. Lancet Neurol.

[2] Chio A, Mazzini L, D'Alfonso S, Corrado L, Canosa A, Moglia C, Manera U, Bersano E, Brunetti M, Barberis M (2018). The multistep hypothesis of ALS revisited: the role of genetic mutations. Neurology.

[3] Al-Chalabi A, van den Berg LH, Veldink J (2017). Gene discovery in amyotrophic lateral sclerosis: implications for clinical management. Nat Rev Neurol.

[4] Chia R, Chio A, Traynor BJ (2018). Novel genes associated with amyotrophic lateral sclerosis: diagnostic and clinical implications. Lancet Neurol.

[5] Xue YC, Ng CS, Xiang P, Liu H, Zhang K, Mohamud Y, Luo H (2020). Dysregulation of RNA-binding proteins in amyotrophic lateral sclerosis. Front Mol Neurosci.

[6] Bozzoni V, Pansarasa O, Diamanti L, Nosari G, Cereda C, Ceroni M (2016). Amyotrophic lateral sclerosis and environmental factors. Funct Neurol.

[7] Yu B, Pamphlett R (2017). Environmental insults: critical triggers for amyotrophic lateral sclerosis. Transl Neurodegener.

[8] Kury P, Nath A, Creange A, Dolei A, Marche P, Gold J, Giovannoni G, Hartung HP, Perron H (2018). Human endogenous retroviruses in neurological diseases. Trends Mol Med.

[9] Xue YC, Feuer R, Cashman N, Luo H (2018). Enteroviral infection: the forgotten link to amyotrophic lateral sclerosis?. Front Mol Neurosci.

[10] Douville R, Liu J, Rothstein J, Nath A (2011). Identification of active loci of a human endogenous retrovirus in neurons of patients with amyotrophic lateral sclerosis. Ann Neurol.

[11] Douville RN, Nath A (1986). Human endogenous retrovirus-K and TDP-43 expression bridges ALS and HIV neuropathology. Front Microbiol.

[12] Li W, Lee MH, Henderson L, Tyagi R, Bachani M, Steiner J, Campanac E, Hoffman DA, von Geldern G, Johnson K (2015). Human endogenous retrovirus-K contributes to motor neuron disease. Sci Transl Med.

[13] Garcia-Montojo M, Fathi S, Norato G, Smith BR, Rowe DB, Kiernan MC, Vucic S, Mathers S, van Eijk RPA, Santamaria U (2021). Inhibition of HERV-K (HML-2) in amyotrophic lateral sclerosis patients on antiretroviral therapy. J Neurol Sci.

[14] Gold J, Rowe DB, Kiernan MC, Vucic S, Mathers S, van Eijk RPA, Nath A, Garcia Montojo M, Norato G, Santamaria UA (2019). Safety and tolerability of Triumeq in amyotrophic lateral sclerosis: the Lighthouse trial. Amyotroph Lateral Scler Frontotemporal Degener.

[15] Huang HI, Shih SR (2015). Neurotropic enterovirus infections in the central nervous system. Viruses.

[16] Rhoades RE, Tabor-Godwin JM, Tsueng G, Feuer R (2011). Enterovirus infections of the central nervous system. Virology.

[17] Feuer R, Ruller CM, An N, Tabor-Godwin JM, Rhoades RE, Maciejewski S, Pagarigan RR, Cornell CT, Crocker SJ, Kiosses WB (2009). Viral persistence and chronic immunopathology in the adult central nervous system following Coxsackievirus infection during the neonatal period. J Virol.

[18] Han J, Ma XJ, Wan JF, Liu YH, Han YL, Chen C, Tian C, Gao C, Wang M, Dong XP (2010). Long persistence of EV71 specific nucleotides in respiratory and feces samples of the patients with Hand-Foot-Mouth Disease after recovery. BMC Infect Dis.

[19] Julien J, Leparc-Goffart I, Lina B, Fuchs F, Foray S, Janatova I, Aymard M, Kopecka H (1999). Postpolio syndrome: poliovirus persistence is involved in the pathogenesis. J Neurol.

[20] Berger MM, Kopp N, Vital C, Redl B, Aymard M, Lina B (2000). Detection and cellular localization of enterovirus RNA sequences in spinal cord of patients with ALS. Neurology.

[21] Giraud P, Beaulieux F, Ono S, Shimizu N, Chazot G, Lina B (2001). Detection of enteroviral sequences from frozen spinal cord samples of Japanese ALS patients. Neurology.

[22] Woodall CJ, Riding MH, Graham DI, Clements GB (1994). Sequences specific for enterovirus detected in spinal cord from patients with motor neurone disease. BMJ.

[23] Vandenberghe N, Leveque N, Corcia P, Brunaud-Danel V, Salort-Campana E, Besson G, Tranchant C, Clavelou P, Beaulieux F, Ecochard R (2010). Cerebrospinal fluid detection of enterovirus genome in ALS: a study of 242 patients and 354 controls. Amyotroph Lateral Scler.

[24] Nix WA, Berger MM, Oberste MS, Brooks BR, McKenna-Yasek DM, Brown RH, Roos RP, Pallansch MA (2004). Failure to detect enterovirus in the spinal cord of ALS patients using a sensitive RT-PCR method. Neurology.

[25] Swanson NR, Fox SA, Mastaglia FL (1995). Search for persistent infection with poliovirus or other enteroviruses in amyotrophic lateral sclerosis-motor neurone disease. Neuromuscul Disord.

[26] Walker MP, Schlaberg R, Hays AP, Bowser R, Lipkin WI (2001). Absence of echovirus sequences in brain and spinal cord of amyotrophic lateral sclerosis patients. Ann Neurol.

[27] Cermelli C, Vinceti M, Beretti F, Pietrini V, Nacci G, Pietrosemoli P, Bartoletti A, Guidetti D, Sola P, Bergomi M (2003). Risk of sporadic amyotrophic lateral sclerosis associated with seropositivity for herpesviruses and echovirus-7. Eur J Epidemiol.

[28] Brooks SP, Dunnett SB (2009). Tests to assess motor phenotype in mice: a user's guide. Nat Rev Neurosci.

[29] Knippenberg S, Thau N, Dengler R, Petri S (2010). Significance of behavioural tests in a transgenic mouse model of amyotrophic lateral sclerosis (ALS). Behav Brain Res.

[30] Deacon RM. Measuring the strength of mice. J Vis Exp. 2013. 10.3791/2610 PMC3725666 23770643

[31] White JJ, Arancillo M, Stay TL, George-Jones NA, Levy SL, Heck DH, Sillitoe RV (2014). Cerebellar zonal patterning relies on Purkinje cell neurotransmission. J Neurosci.

[32] Ruifrok AC, Johnston DA (2001). Quantification of histochemical staining by color deconvolution. Anal Quant Cytol Histol.

[33] Bruijn LI, Becher MW, Lee MK, Anderson KL, Jenkins NA, Copeland NG, Sisodia SS, Rothstein JD, Borchelt DR, Price DL, Cleveland DW (1997). ALS-linked SOD1 mutant G85R mediates damage to astrocytes and promotes rapidly progressive disease with SOD1-containing inclusions. Neuron.

[34] Gurney ME, Pu H, Chiu AY, Dal Canto MC, Polchow CY, Alexander DD, Caliendo J, Hentati A, Kwon YW, Deng HX (1994). Motor neuron degeneration in mice that express a human Cu, Zn superoxide dismutase mutation. Science.

[35] Xue YC, Ruller CM, Fung G, Mohamud Y, Deng H, Liu H, Zhang J, Feuer R, Luo H (2018). Enteroviral infection leads to transactive response DNA-binding protein 43 pathology in vivo. Am J Pathol.

[36] Beers DR, Appel SH (2019). Immune dysregulation in amyotrophic lateral sclerosis: mechanisms and emerging therapies. Lancet Neurol.

[37] McCauley ME, Baloh RH (2019). Inflammation in ALS/FTD pathogenesis. Acta Neuropathol.

[38] Thonhoff JR, Simpson EP, Appel SH (2018). Neuroinflammatory mechanisms in amyotrophic lateral sclerosis pathogenesis. Curr Opin Neurol.

[39] Butovsky O, Siddiqui S, Gabriely G, Lanser AJ, Dake B, Murugaiyan G, Doykan CE, Wu PM, Gali RR, Iyer LK (2012). Modulating inflammatory monocytes with a unique microRNA gene signature ameliorates murine ALS. J Clin Invest.

[40] Jara JH, Genc B, Stanford MJ, Pytel P, Roos RP, Weintraub S, Mesulam MM, Bigio EH, Miller RJ, Ozdinler PH (2017). Evidence for an early innate immune response in the motor cortex of ALS. J Neuroinflamm.

[41] Nivon M, Fort L, Muller P, Richet E, Simon S, Guey B, Fournier M, Arrigo AP, Hetz C, Atkin JD, Kretz-Remy C (2016). NFkappaB is a central regulator of protein quality control in response to protein aggregation stresses via autophagy modulation. Mol Biol Cell.

[42] Whitton JL, Cornell CT, Feuer R (2005). Host and virus determinants of picornavirus pathogenesis and tropism. Nat Rev Microbiol.

[43] Ruller CM, Tabor-Godwin JM, Van Deren DA, Robinson SM, Maciejewski S, Gluhm S, Gilbert PE, An N, Gude NA, Sussman MA (2012). Neural stem cell depletion and CNS developmental defects after enteroviral infection. Am J Pathol.

[44] Fung G, Shi J, Deng H, Hou J, Wang C, Hong A, Zhang J, Jia W, Luo H (2015). Cytoplasmic translocation, aggregation, and cleavage of TDP-43 by enteroviral proteases modulate viral pathogenesis. Cell Death Differ.

[45] Ling SC, Polymenidou M, Cleveland DW (2013). Converging mechanisms in ALS and FTD: disrupted RNA and protein homeostasis. Neuron.

[46] Garofalo S, Cocozza G, Porzia A, Inghilleri M, Raspa M, Scavizzi F, Aronica E, Bernardini G, Peng L, Ransohoff RM (2020). Natural killer cells modulate motor neuron-immune cell cross talk in models of Amyotrophic Lateral Sclerosis. Nat Commun.

[47] Gustafson MP, Staff NP, Bornschlegl S, Butler GW, Maas ML, Kazamel M, Zubair A, Gastineau DA, Windebank AJ, Dietz AB (2017). Comprehensive immune profiling reveals substantial immune system alterations in a subset of patients with amyotrophic lateral sclerosis. PLoS ONE.

[48] Lewis CA, Manning J, Rossi F, Krieger C (2012). The neuroinflammatory response in ALS: the roles of microglia and T cells. Neurol Res Int.

[49] Mammana S, Fagone P, Cavalli E, Basile MS, Petralia MC, Nicoletti F, Bramanti P, Mazzon E (2018). The role of macrophages in neuroinflammatory and neurodegenerative pathways of Alzheimer's disease, amyotrophic lateral sclerosis, and multiple sclerosis: pathogenetic cellular effectors and potential therapeutic targets. Int J Mol Sci.

[50] Naor S, Keren Z, Bronshtein T, Goren E, Machluf M, Melamed D (2009). Development of ALS-like disease in SOD-1 mice deficient of B lymphocytes. J Neurol.

[51] Upasani V, Rodenhuis-Zybert I, Cantaert T (2021). Antibody-independent functions of B cells during viral infections. PLoS Pathog.

[52] Mackenzie IR, Bigio EH, Ince PG, Geser F, Neumann M, Cairns NJ, Kwong LK, Forman MS, Ravits J, Stewart H (2007). Pathological TDP-43 distinguishes sporadic amyotrophic lateral sclerosis from amyotrophic lateral sclerosis with SOD1 mutations. Ann Neurol.

[53] Robertson J, Sanelli T, Xiao S, Yang W, Horne P, Hammond R, Pioro EP, Strong MJ (2007). Lack of TDP-43 abnormalities in mutant SOD1 transgenic mice shows disparity with ALS. Neurosci Lett.

[54] Ayers JI, Cashman NR (2018). Prion-like mechanisms in amyotrophic lateral sclerosis. Handb Clin Neurol.

[55] Frost B, Diamond MI (2010). Prion-like mechanisms in neurodegenerative diseases. Nat Rev Neurosci.

[56] Brown RH, Al-Chalabi A (2017). Amyotrophic lateral sclerosis. N Engl J Med.

[57] Hardiman O, Al-Chalabi A, Chio A, Corr EM, Logroscino G, Robberecht W, Shaw PJ, Simmons Z, van den Berg LH (2017). Amyotrophic lateral sclerosis. Nat Rev Dis Primers.

