# Supplementary figures and images for: Sublethal enteroviral infection exacerbates disease progression in an ALS mouse model
Source: J Neuroinflammation. 2022 Jan 12;19:16. doi: 10.1186/s12974-022-02380-7 (PMC8753920; doi:10.1186/s12974-022-02380-7)

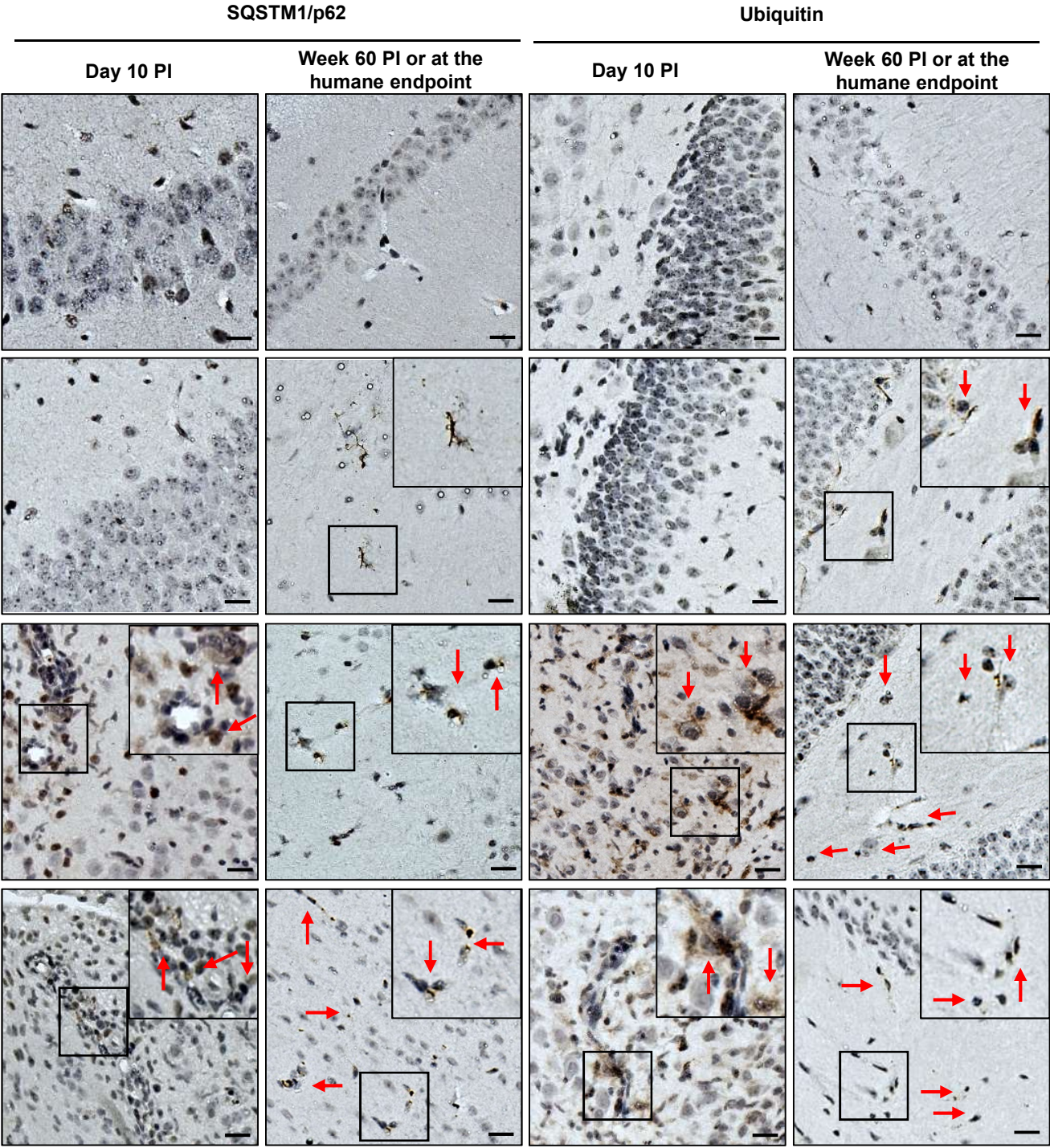

Supplement: Supplementary file 1 — Additional file 1: Fig. S1. Sublethal CVB3 infection leads to ALS-related pathologies in vivo. Representative images of SQSTM1/p62 and ubiquitin immunohistochemical staining in the hippocampus regions of the brain from mock- and CVB3-injected C57BL/6J or SOD1G85R mice at day 10 PI or week 60 PI (or humane endpoint) as indicated. The red arrows indicate SQSTM1/p62 or ubiquitin positive inclusions. Black boxes on the top right illustrate the enlarged images. Scale bar = 100 μm. [file 12974_2022_2380_MOESM1_ESM.pdf]

Additional file 2: Fig. S2

**A** Day 10 PI

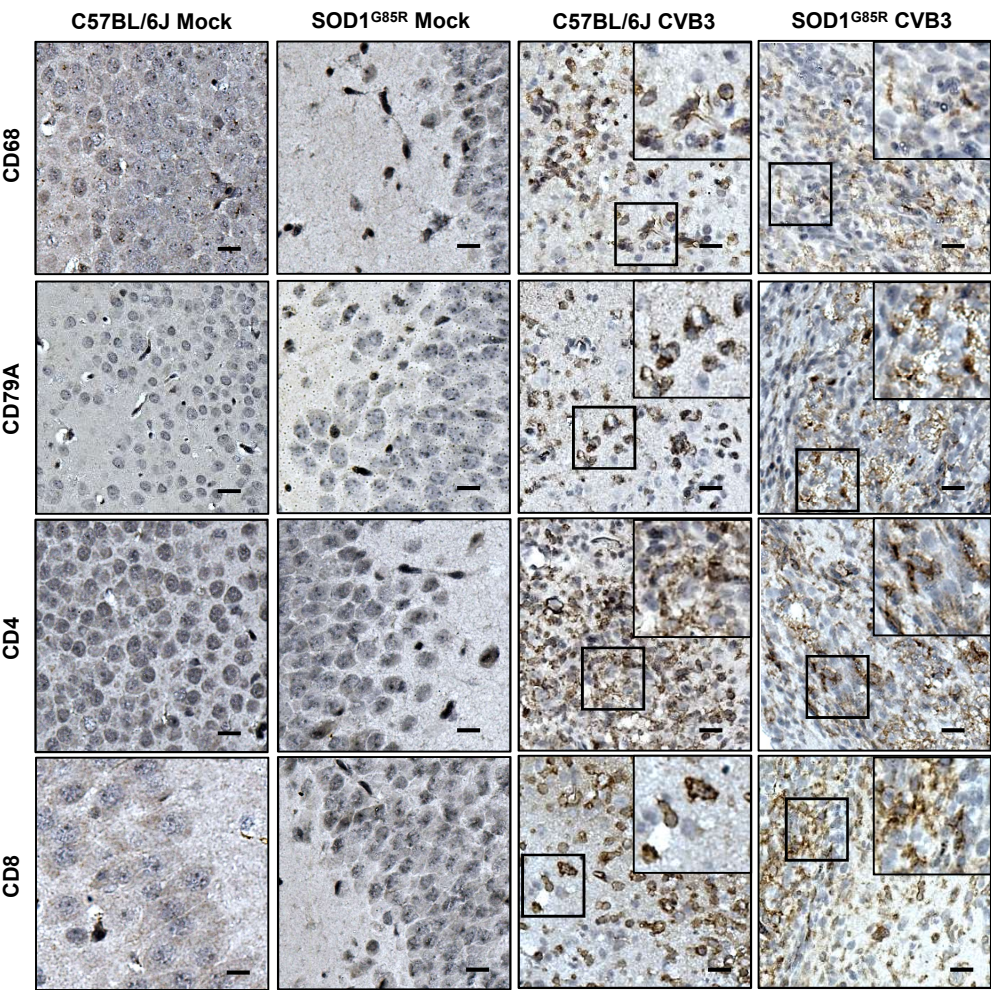

**B** Week 60 PI or at the humane endpoint

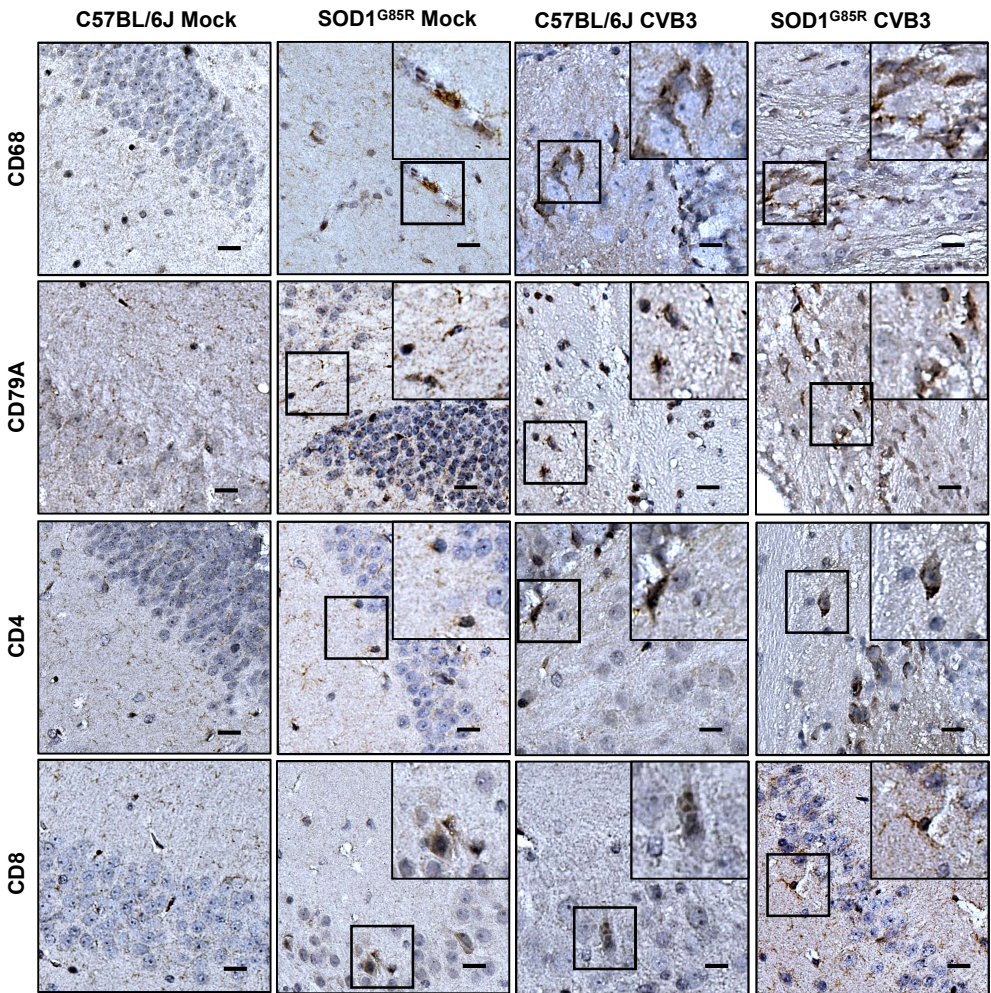

Supplement: Supplementary file 2 — Additional file 2: Fig. S2. Sublethal CVB3 infection triggers microglia/astrocyte activation and immune infiltration in mice. Representative images of CD68, CD79A, CD4 and CD8 immunohistochemical staining in the hippocampus regions of the brain from mock- and CVB3-infected C57BL/6J or SOD1G85R mice at day 10 PI (A) or week 60 PI (or humane endpoint) (B). Similar observation was made in the regions of infected cerebral cortex (data not shown). Immune infiltrations were quantified by optical density based on the IHC staining in the hippocampus and cerebral cortex regions. Black boxes on the top right illustrate the enlarged images. Scale bar = 100 μm. [file 12974_2022_2380_MOESM2_ESM.pdf]
